# Knowledge and Awareness of Parents Attending Pediatric Clinic Regarding Pediatric Obstructive Sleep Apnea in Jeddah: A Cross-Sectional Study

**DOI:** 10.7759/cureus.35339

**Published:** 2023-02-22

**Authors:** Rayan M Alosaimi, Mohammed T Musslem, Feras F Filfilan, Gutaybah S Alqarni, Essa A Alazmi, Talal Y Alghamdi, Jehad R Alsaedi, Hosam Amoodi

**Affiliations:** 1 Medicine, University of Jeddah, Jeddah, SAU; 2 Otolaryngology-Head and Neck Surgery, University of Jeddah, Jeddah, SAU; 3 Otolaryngology-Head and Neck Surgery, Dr. Soliman Fakeeh Hospital, Jeddah, SAU

**Keywords:** sleep-disordered breathing, a cross-sectional study, jeddah saudi arabia, knowledge & awareness, pediatric obstructive sleep apnea

## Abstract

Background: Obstructive sleep apnea (OSA) is characterized by chronic, recurrent episodes of partial or complete airway obstruction during sleep. It has a negative impact on quality of life and behavior and can lead to adverse neurological and cardiovascular outcomes if left untreated. This study aims to assess the awareness and knowledge of pediatric OSA among parents attending a general pediatric clinic in Jeddah, Saudi Arabia.

Methods: An observational cross-sectional study was conducted from October 2022 to December 2022, on parents who attended the pediatric clinic at Dr. Soliman Fakeeh Hospital in Jeddah. Participants were asked to complete a self-administered questionnaire, either using a tablet or a paper-based survey. The questionnaire consisted of sociodemographic information and questions assessing the parents' knowledge and awareness of pediatric OSA.

Results: The study included 146 participants. The mean knowledge score was 15.38 ± 6. Only 20% of the participants had a good knowledge level, while 80% had a poor level of knowledge. Furthermore, regarding the definition of OSA, 60 out of 146 participants answered correctly. Enlargement of adenoids was the most recognized risk factor, and restless sleep was the most recognized symptom. The majority of participants agreed that consulting an expert doctor was the best method to raise awareness about childhood OSA.

Conclusion: The result of our study reveals the low level of awareness and knowledge of pediatric OSA among parents attending a pediatric clinic in Jeddah. This highlights the need for health education programs and sensitization campaigns to improve awareness of pediatric obstructive sleep apnea.

## Introduction

Obstructive Sleep Apnea (OSA) is a sleep-related breathing disorder characterized by chronic recurrent episodes of partial or complete airway obstruction [1]. Children with OSA suffer from intermittent hypercapnia, hypoxemia, arousal, and sleep fragmentation, which negatively impact their quality of life and behavior and can lead to several adverse neurodevelopmental and cardiovascular consequences [2-4].

The prevalence rate of OSA in the pediatric population is estimated to be 1% to 5%, with adenotonsillar hypertrophy being the most common cause [3,4]. Pediatric OSA typically manifests as habitual snoring and cognitive and behavioral symptoms such as learning difficulties, attention deficit, hyperactivity, and impulsivity, which can be linked to the diagnosis of attention deficit hyperactivity disorder (ADHD) [3-5]. Furthermore, pediatric OSA has been linked to developmental consequences and morbidities, including neurocognitive disabilities impacting school performance, behavior, and social development. Depending on its severity, it can also lead to cardiovascular and metabolic morbidities [3,4].

Multiple studies show a correlation between treating pediatric OSA and improvements in cognition, behavior, quality of life and sleep [4-6]. This highlights the importance of parent awareness and understanding of pediatric OSA to facilitate early diagnosis and treatment [7].

In Saudi Arabia, a study involving 1000 participants from the adult population revealed a lack of awareness about OSA [8]. Another study conducted in the Asir region also showed low levels of awareness [9]. The level of awareness regarding pediatric OSA among parents was only assessed in one study involving 675 parents, with nearly one-third found to have lower levels of awareness [10]. Other studies in the dental field have revealed a less-than-optimal understanding of OSA [11-14].

Knowledge and awareness of parents regarding pediatric OSA are crucial for seeking medical attention. This cross-sectional study aims to determine the level of awareness and knowledge of pediatric OSA among parents visiting a pediatric clinic in Jeddah, Saudi Arabia.

## Materials and methods

Design, participants, and setting

This observational cross-sectional study was conducted from October 2022 to December 2022. We recruited parents who attended the pediatric clinic during the weekdays from 10 am to 10 pm at Dr. Soliman Fakeeh Hospital in Jeddah, Saudi Arabia. Participants were recruited through convenience sampling. After obtaining permission, parents were asked to complete the self-administered questionnaire using a tablet or a paper-based survey. We included parents of any age, nationality, educational level, or occupation.

Questionnaire

We adapted a previously published questionnaire with permission [10]. The questionnaire was modified to meet our study objectives. A statistical expert was engaged who did a pilot study on 24 participants to test the reliability and validity of the questionnaire. The Cronbach's alpha value was 0.82. The questionnaire consisted of two parts: questions about sociodemographic characteristics and questions to evaluate parental knowledge and awareness of pediatric OSA, including an understanding of its definition, general information, symptoms, and risk factors. The scoring system contained 30 questions. The test was scored based on responses to the second part of the questionnaire. According to the total score, we categorized the participants into two groups: a good knowledge group with a score of >70%, and a poor knowledge group with a score of ≤70%. One last question asked the parents to indicate what is the best way that could help to improve the community awareness of childhood OSA.

Data collection and statistical analysis

Data collection and management were completed by using Google Forms (Google LLC, Mountain View, CA, USA) and Microsoft Office Excel (Microsoft Corp., Redmond, WA, USA). Continuous data were represented as means and standard deviation, while categorical data were described in frequencies and percentages using tables and figures. To assess the variation in mean knowledge scores across the sociodemographic characteristics we used a one-way analysis of variance (ANOVA) test. The statistical significance of the p-value was defined as ≤0.05, with a confidence interval of 95%. The statistical analysis was performed using SPSS version 29 (IBM Corp., Armonk, NY, USA).

Ethical consideration

The study was approved by the Institutional Review Board at Dr. Soliman Fakeeh Hospital, Jeddah, Saudi Arabia (approval no. 368/IRB/2022). The first page of the questionnaire contained written consent stating that by completing this questionnaire the participant agreed to participate in this study.

## Results

**Figure 1:**
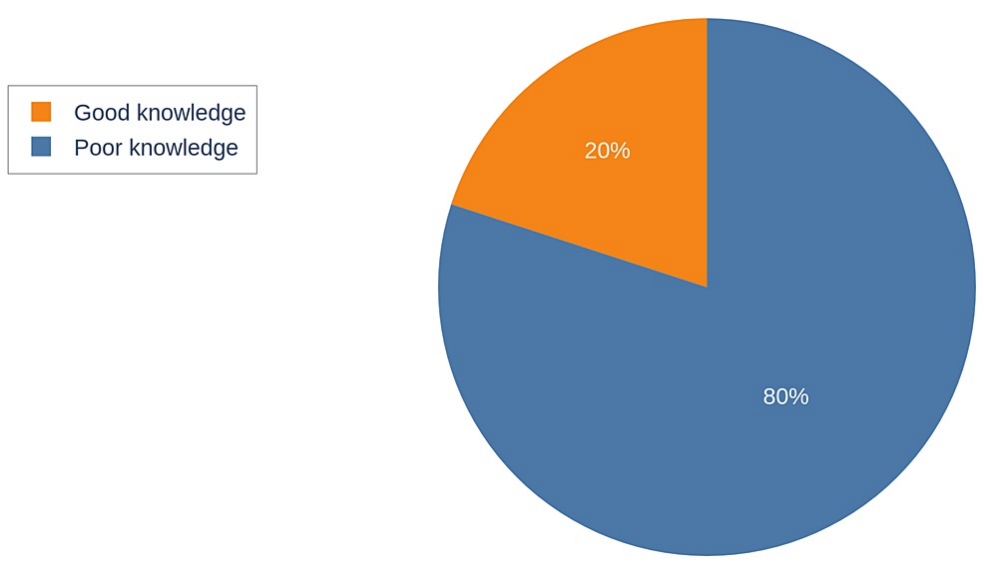
Pie chart demonstrating the percentage of participants' knowledge level

A total of 146 participants were included in this study. Table 1 demonstrates the sociodemographic characteristics of the participants. The majority were married Saudi parents in the age group of 31 to 40. The gender distribution was nearly equal. Most were highly educated, with a bachelor's degree or higher. The number of participants' children was predominantly one to three. Additionally, 19% of the parents claimed that they had at least one child affected with OSA. The participants' mean knowledge score was 15.38 ± 6. A one-way ANOVA test revealed a statistically significant difference in mean knowledge scores across gender (p=0.05) and the number of children (p=0.032). As shown in Figure 1, only 20% of participants had a good level of knowledge, while the remaining 80% had poor knowledge, as determined by their total knowledge score.

**Table 1:** Sociodemographic characteristics and mean knowledge score by sociodemographic variables

Characteristics		Number of participants (%)	Knowledge score (mean ± standard deviation)	p-value
Gender	Male	75 (51.4)	14.420 ± 6.48	.050
	Female	71 (48.6)	16.394 ± 5.48	
Age group (years)	18-30	34 (23.3)	15.70 ± 5.59	.297
	31-40	73 (50.0)	14.74 ± 5.92	
	41-50	33 (22.6)	15.75 ± 7.07	
	51-60	4 (2.7)	21.37 ± 1.88	
	60+	2 (1.4)	14.75 ± 3.88	
Nationality	Saudi	97 (66.4)	14.85 ± 5.51	.143
	Non-Saudi	49 (33.6)	16.41 ± 7.01	
Marital status	Married	142 (97.3)	15.33 ± 6.12	.591
	Divorced	4 (2.7)	18.66 ± 4.04	
Education	High school degree and below	34 (23.3)	14.90 ± 6.06	.118
	Undergraduate	6 (4.1)	20.00 ± 4.38	
	Higher education	15 (10.3)	17.4± 5.38	
	Bachelor	91 (62.3)	14.95± 6.2	
Occupation	Health sector employee	10 (6.8)	16.8 ± 6.39	.663
	Engineer	19 (13)	14.158 ± 7.34	
	Teacher	5 (3.4)	14.000 ± 4.35	
	Soldier	8 (5.5)	17.375 ± 6.04	
	Other	104 (71.2)	15.380 ± 5.91	
Number of children	1-3	114 (78.1)	14.68 ± 5.93	.032
	4-7	30 (20.5)	17.9 ± 6.21	
	More than 8	2 (1.4)	17.25 ± 3.88	
Knowledge score		146 (100)	15.38 ± 6	

Most of the participants stated that they knew about childhood OSA, their source of information was from social media (18.7%), medical articles (17.7%), a person affected with OSA (12.3%), and other sources of information (13.8%). Around 37.4% had never heard about childhood OSA. Regarding the definition of OSA only 60 out of 146 participants answered correctly, while the remaining majority didn’t know what OSA is.

Table 2 shows the results of general knowledge questions, with only three questions being answered correctly by the majority of participants: 82.9% agreed that childhood OSA can be managed, 85.6% recognized that early intervention and management can reduce the risks of complications, and 85.6% believed that parents' awareness about childhood OSA can help reduce the burden on them and the population.

**Table 2:** Percentages and frequencies of general knowledge answers

General knowledge items		Frequency	Percentage
Do you know that childhood obstructive sleep apnea can affect a child’s school performance?	No	81	55.5%
	Yes	65	44.5%
Do you know that children with obstructive sleep apnea have a higher prevalence of depression than other children?	No	92	63.0%
	Yes	54	37.0%
Do you know that childhood obstructive sleep apnea affects attention and behavior?	No	77	52.7%
	Yes	69	47.3%
Do you think that the genetic factor can have a role in the cause of childhood obstructive sleep apnea?	No	86	58.9%
	Yes	60	41.1%
Do you think that childhood obstructive sleep apnea can be managed?	No	25	17.1%
	Yes	121	82.9%
Do you think early intervention and management can reduce the possible risk of complications in children with obstructive sleep apnea?	No	21	14.4%
	Yes	125	85.6%
Do you think parents' awareness about childhood obstructive sleep apnea can help reduce the burden on them and the population?	No	21	14.4%
	Yes	125	85.6%

**Figure 2:**
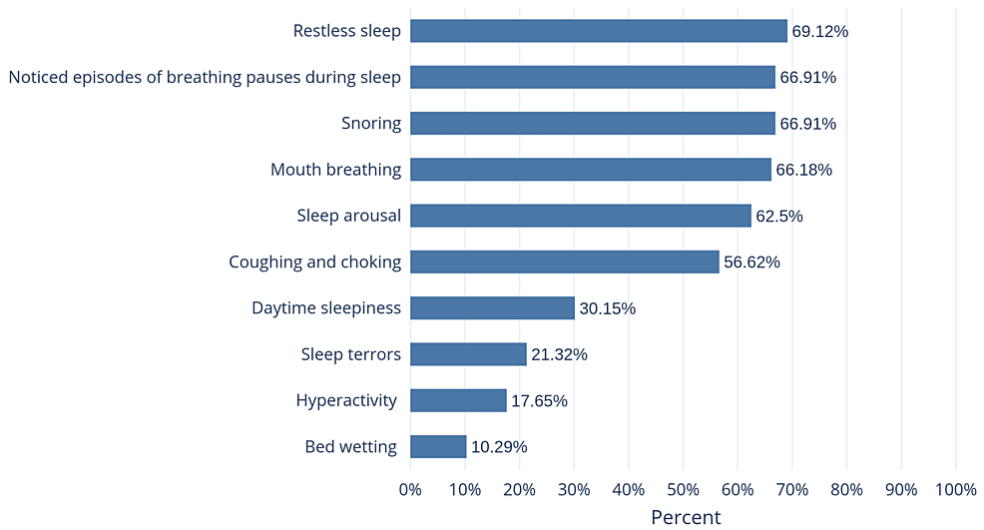
The percentage of correct answers regarding the symptoms of childhood OSA OSA: Obstructive sleep apnea

Figure 2 shows the frequencies of correct answers regarding the symptoms of childhood OSA, with restless sleep being the most recognized symptom, followed by snoring and noticeable episodes of breathing pauses during sleep. Meanwhile, daytime sleepiness, sleep terrors, hyperactivity, and bed wetting were the least recognized symptoms.

**Figure 3:**
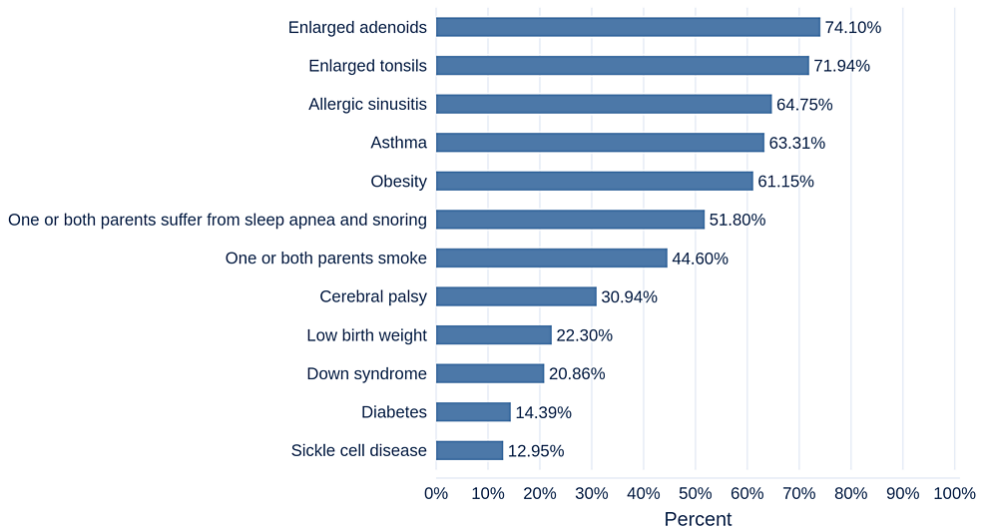
The percentage of correct answers regarding the risk factors of childhood OSA OSA: Obstructive sleep apnea

Figure 3 shows participants' knowledge about the risk factors of childhood OSA, with the most recognized being enlarged adenoids followed by enlarged tonsils. Nearly two-thirds of participants agreed that allergic sinusitis, asthma, and obesity are risk factors for pediatric OSA, while the least recognized risk factors were diabetes and sickle cell anemia.

Regarding the best methods to raise parents’ awareness about childhood OSA, the majority agreed on consulting an expert doctor (39%), followed by volunteer awareness campaigns (34%), and lastly, internet and social media (26%).

## Discussion

As pediatric OSA has a relatively high prevalence of up to 5%, not to mention the serious consequences, we believe that paternal knowledge is essential for the early diagnosis and treatment of pediatric OSA [4]. The present study aimed to assess parental knowledge and awareness of pediatric OSA, making it one of the first studies to focus on parents in Saudi Arabia.

Our findings show a lack of knowledge of pediatric OSA among parents visiting a general pediatric clinic. The mean knowledge score was 15.38 ± 6. Only 20% of the participants had a good knowledge level and only 41% of the parents knew the correct definition of OSA. These results are similar to the findings of a study by Bashir et al., which was the only study that assessed parents’ knowledge regarding pediatric OSA in Saudi Arabia. They concluded that nearly one-third of their participants had a low level of knowledge, considering that they categorized their knowledge level into low, medium, and high, only 6.2% were found to have high knowledge [10]. A similar pattern of limited knowledge and awareness was found in China, where Xu et al. evaluated the knowledge of 1123 parents and found limited knowledge and awareness regarding OSA treatment and complications [15].

Surprisingly, despite having a good education, the majority of the participants had poor knowledge of pediatric OSA. This finding is consistent with the studies by Bashir et al. and Xu et al. [10,15]. However, our study did not find a significant statistical difference in the knowledge scores between different educational levels, which contrasts the results of the previously mentioned studies.

In terms of comparison between mothers and fathers, our study found that mothers had higher knowledge scores than fathers, this was also observed in the study by Xu et al. They found that mothers had significantly higher scores than fathers regarding the symptoms and complications of pediatric OSA, but not treatment [15]. One possible explanation for this finding could be the increased responsibility and involvement of mothers in childcare. Additionally, we found that parents with more than three children had significantly higher knowledge than those with fewer children. This could be due to their exposure as a result of a higher likelihood of prior sleep disorders, including OSA.

In our study, hyperactivity and bed wetting were the least recognized symptoms among the parents. These results are similar to the findings of DiNardo et al. and Bashir et al. [10,16]. On the other hand, adenoids and tonsils enlargement, allergic sinusitis, asthma, and obesity were well-recognized risk factors by the parents in our study. However, in the study by Bashir et al., obesity was poorly recognized as a risk factor [10].

Despite their limited knowledge, most parents agreed that OSA can be managed and early intervention can reduce complications. However, without adequate knowledge about symptoms and risk factors, parents may have difficulty identifying OSA early, leading to delayed diagnosis and treatment. Therefore, it is important to raise parental awareness to ensure early medical intervention, thus limiting complications and reducing the burden of the disease.

In our study, most participants learned about OSA from social media and medical articles. However, they believed that the best method to increase awareness would be to consult with a specialist doctor, followed by awareness campaigns and social media. This information may be useful for decision-makers and healthcare providers in determining the most effective approach to increasing parental knowledge and awareness of pediatric OSA.

Future studies with larger sample sizes and in other regions of Saudi Arabia are necessary to confirm our findings. This study has some limitations, including the small sample size which may limit the generalizability of the results, and the use of a self-reported survey which is subject to recall and desirability biases.

## Conclusions

Our study highlights the pressing need for increased knowledge and awareness among parents regarding pediatric OSA. The results indicate that the majority of parents who attended the pediatric clinic in Jeddah had limited knowledge about the symptoms and risk factors of pediatric OSA. To ensure early diagnosis and treatment of this condition, further efforts to educate parents and raise awareness are crucial. This will help minimize the burden of this disease and prevent its associated complications. Future studies with a larger sample size and in other regions of Saudi Arabia should be conducted to further understand and address the knowledge gap among parents.
